# Gendered activities: Men farm, women trade, or is it less clear-cut?

**DOI:** 10.1371/journal.pone.0332100

**Published:** 2026-01-27

**Authors:** Michael Olabisi, Michael Ogundipe

**Affiliations:** 1 Department of Community Sustainability, Michigan State University, East Lansing, Michigan, United States of America; 2 Department of Agricultural Food and Resource Economics, Michigan State University, East Lansing, Michigan, United States of America; 3 Department of Global Studies, University of California Santa Barbara, California, United States of America; North Carolina State University, UNITED STATES OF AMERICA

## Abstract

The study explores gender participation in agricultural value chains, showing how roles are differentiated across genders and regions. The study fills an important gap in the literature which takes gender disparities as uniform and ignores subnational differences in farming and trading activities, with a focus on Africa’s most populous country, Nigeria. We estimated gender differences in participation, and compared welfare outcomes, using a combination of logit regression models, and instrumental variable estimation, that control for unobserved factors in the model. Household consumption expenditure is used as a proxy for welfare in the data, the most recent General Household Survey, covering more than 32,000 individuals. We find that men dominate farming, while women tend to dominate retail and wholesale trade, but with large regional differences that blur these distinctions. Being a woman implies a lower probability of farming, but almost doubles the likelihood of being engaged in wholesale and about triples that of retailing compared to being a man. The regional differences matter. In the southern regions of the country, women are more diversified across farming and trade activities relative to the north. In assessing the impact of the allocation of activities by gender, we show that farming households spend the least, while households with wholesalers have significantly higher expenditures.

## Introduction

Gender disparities in labor force participation continue to matter in high- and low-income economies. The disparities matter for household welfare in most developing economies, where agriculture remains the primary source of income, and the division of labor between men and women is unequal, and shaped by different cultural norms, access to resources, and shifts in market dynamics [1,2]. Women still grapple with reduced access to land and agricultural inputs, and less control over financial resources that are crucial for farming or trading farm products [3,4]. One open question that results from these disparities, is how much they create differences in the shares of men and women who farm, or who trade farm products.

A robust body of research papers captures the differences in gender participation by profession in several developing economies [5–8]. The differences in participation matter, as they affect household income [9,10]. In developing economies where a majority of households are involved in agriculture, it is important to consider how gender differences feature in different geographic regions and at various stages of the food value chain.

There is a remarkable gap in this literature: the absence of studies that document regional and intra-national differences in gender-participation gaps. Furthermore, looking at how regional differences in gender participation evolve through the stages of food value chains promises new insights. Most of the literature presents gender gaps as nationally uniform, overlooking how specific subnational cultural patterns may shape the most salient outcomes [1,2,11]. On the contrary, a growing literature shows that these sub-national differences matter, either in infrastructure [12,13], or market access and sociocultural norms [14]. The oversight by researchers here, hides the lived experiences of women and men whose roles in agriculture are spread unevenly across different sub-national units and regions.

Women’s participation in agricultural value chains does not automatically improve their economic outcomes. There are documented differences in scale by gender for example, that matter for household outcomes [15]. Even though women participate in market-linked agricultural chains-segments beyond on-farm production that connect outputs to buyers (processing, transport, wholesaling, and retail)—the economic benefits are often shared unequally, with male household members more likely to gain the rewards when women have no control over resources [10]. In northern Nigeria’s tomato corridor, for example, women participate more in retail markets, but men usually control bulk purchasing, price-setting and profits through market associations [16].

This paper contributes the first study (we know of), that documents how gender participation changes from farming to wholesale and retail, at different rates for different sub-regions of a developing economy. Our thesis is that the sub-national differences matter for how national and development policies should be structured, since gendered participation is not uniform but is mediated by geography.

Our paper therefore complements previous articles that show gender differences in participation in farming [2,6,7,17–20]. Our main addition to this literature is showing that gendered divisions extend beyond farm production into wholesale and retail segments and that these effects vary sharply by region. Considering retail and wholesale activities is important, because as we show, incomes from these activities differ notably from farming.

We are one of the first papers to show gender differences in participation in wholesale markets in a developing economy such as Nigeria. Seminal papers on this topic show differences for Kenya [8,21], India [1,22], and Malawi [11]. The innovation in this paper is the use of a nationally representative survey (rather than a market survey) to document differences in the prevalence of gender at different stages of the value chain.

Building on this contribution, we offer a framework that can help guide policy discussions on how to improve women’s access to economic activities both in agriculture and trade. This paper helps to identify where the constraints are most binding and where targeted support may be most effective by investigating the patterns across regions and activities. This same approach could be considered in other settings where gender roles in agriculture and commerce shape household welfare, making this analysis relevant beyond Nigeria.

## Materials and methods

### Data

This study uses data from the Nigeria General Household Survey, a nationally representative living standards measurement survey, which was implemented by the Nigerian National Bureau of Statistics, in collaboration with the World Bank [23]. Data were collected in multiple waves, each wave covering 2 visits: Post-Planting and Post-Harvest. For this study, we focus on wave 5 (2023/24), as it provides the most recent and comprehensive data on labor activities, demography, and agricultural participation. This survey was carried out in the 37 states of Nigeria covering rural and urban households in the six geopolitical zones. We accessed the survey data in July 2025 from the World Bank website. No information from the data could be used to identify individual participants during or after data collection. The wave 5 survey consisted of 4,771 distinct households and 32,009 total individuals.

For our study, the main unit of analysis is the individual, with household-level variables used to contextualize the results. We focused on individuals reporting primary participation in agricultural value-chain activities or specific non-farm sales activities, given the importance of agriculture to the economy [24]. We included only respondents with complete demographic and labor activity information. This targeted focus allowed us to explore gender and regional differences throughout the value chain.

We organized all reported activities into four categories: Farming, Agricultural Labor, Wholesale and Retail Trade, and ‘Other’, for all other occupations.

### Empirical strategy

Our analysis proceeds in two stages. First, we examine the factors that shape an individual’s likelihood of working in farming, wholesaling, or retailing. Second, we study how these activity choices relate to household welfare. To do this, we use three models: a logit model, ordinary least squares (OLS), and an instrumental variable (IV) model.

The dependent variables for participation in farming, wholesale, and retail take the value 1 for participation and 0 otherwise. Therefore, we use a logit model to estimate the probability that an individual engages in each activity while controlling for personal and household characteristics. The logit estimator is usually more robust to outliers, and we do not want to presume that our latent variable has a normal distribution. This allows us to examine how gender, education, location, and other factors shape participation in different parts of the value chain. The inclusion of these variables is supported by theory, as well as past research showing that the choices and opportunities for work are shaped by the variables [e.g., [2,4,10].

For individual *i* in region *r*, the probability of engaging in activity *j* is modeled as:

$$\mathrm{Pr}(Y_{ij}=1)=\frac{\exp\left(\beta_{0}+\beta_{1}{\text{Female}}_{i}+\beta_{2}{𝐗}_{i}+\beta_{3}{𝐙}_{r}\right)}{1+\exp\left(\beta_{0}+\beta_{1}{\text{Female}}_{i}+\beta_{2}{𝐗}_{i}+\beta_{3}{𝐙}_{r}\right)},$$ (1)

Where *Y*_*ij*_ denotes participation in farming, wholesale, or retail trade; ${𝐗}_{i}$ includes gender, sector, age, education, marital status, land ownership, house ownership; and ${𝐙}_{r}$ are regional dummies. Gender is a categorical variable and takes 0 for male and 1 for female. The age variable is a categorical variable that takes the following values: less than 35 years, 35–50 years, 51–65 years, and above 65. The education variable is a dummy that captures whether the respondent has ever attended school (Yes = 1, No = 0). Marital status is a categorical variable—Married, Never Married, and Others. Land ownership is a dummy variable equal to 1 if the household owns land and 0 otherwise. The rural–urban indicator is a dummy variable equal to 1 for rural residents and 0 for urban residents. Zone dummies are also included, covering North Central, North East, North West, South East, South South, and South West.

To assess welfare differences, we use total household consumption expenditure as the outcome variable. OLS provides a simple way to compare the spending levels of households that engage in different activities.

Household welfare is estimated as:

$$C_{h}=\alpha_{0}+\alpha_{1}{\text{Activity}}_{h}+\alpha_{2}{𝐗}_{h}+\mu_{h},$$ (2)

Where *C*_*h*_ denotes household consumption expenditure, ${\text{Activity}}_{h}$ captures the household’s main value-chain engagement (farming only, retail only, wholesale only, or combinations), and ${𝐗}_{h}$ includes household characteristics.

However, because occupational choice may be endogenous, and may be influenced by unobserved factors that are not captured in the data, we also estimate an IV model to obtain more reliable estimates of the effect of activity choice on household welfare. Our instruments are the averages for the locality or enumeration area of each household of occupational categories. The IV approach helps us separate the effect of activity choice from the unobserved influences that usually make activities clustered by area.

The IV model takes the form:

$$\text{First stage:}\;{\text{Activity}}_{h}=\gamma_{0}+\gamma_{1}{\text{Instrument}}_{h}+\gamma_{2}{𝐗}_{h}+\varepsilon_{h},$$ (3)

$$\text{Second stage:}\;C_{h}=\alpha_{0}+\alpha_{1}{\hat{\text{Activity}}}_{h}+\alpha_{2}{𝐗}_{h}+\mu_{h},$$ (4)

Where ${\text{Instrument}}_{h}$ is an exogenous factor correlated with activity choice, in this case, the average activity choice of other households in the area, but not directly with household expenditure. This approach allows us to isolate the welfare impact of participating in different value-chain activities.

Table 1 summarizes the variables used in the estimation steps, and how they contribute to the estimations. The category with the value zero (0) served as the baseline for each categorical variable, except for the marital status variable, for which we used the category labeled ‘Others’–the category with widowed, separated or divorced persons, as the baseline. We used the North Central zone as the baseline for geo-political zone categories in Table 5.

**Table 1:** Definitions of variables used in the models.

Variable	Role(s)	Description	Coding
Farming participation	Dependent (logit)	Individual works on own or family farm	Dummy: 1 = participates,eak 0 = otherwise
Wholesale participation	Dependent (logit)	Individual works in wholesale trade	Dummy: 1 = participates,eak 0 = otherwise
Retail participation	Dependent (logit)	Individual works in retail trade	Dummy: 1 = participates,eak 0 = otherwise
Household consumption	Dependent (OLS, IV)	Total household expenditure (welfare measure)	Continuous (Naira)
Gender	Independent	Respondent is female	Dummy: 1 = female, 0 = male
Sector	Independent	Location of residence	Dummy: 1 = rural, 0 = urban
Education	Independent	Ever attended school	Dummy: 1 = no schooling,eak 0 = attended school
Age group	Independent	Age category of respondent	<35; 35–50; 51–65; >65
Marital status	Independent	Respondent’s marital category	Married; Never married; Others
Land ownership	Independent	Household owns agricultural land	Dummy: 1 = owns land,eak 0 = Does not own land
House ownership	Independent	Household owns dwelling unit	Dummy: 1 = owns house,eak 0 = does not own house
Zone	Independent	Nigeria’s geopolitical zone	0 = North Central; 1 = North East; 2 = North West; 3 = South East; 4 = South South;eak 5 = South West
Average activity type	Independent (OLS, IV)	Households’ main activity, averaged by enumeration area	Seven binary variables indicating: farmers only; retailers only; wholesalers only; farmers + retailers; farmers + wholesalers; wholesalers + retailers; farmers + wholesalers + retailers.

## Results

### Descriptive statistics

Table 2 highlights the gendered share of activities participation in agricultural value chain in Nigeria. Men dominate farming (61.1%) and agricultural labor (76.5%), while women dominate retailing (74.4%) and wholesaling (65.8%). These figures show that women are more engaged in sales and trading roles, suggesting gendered division of labor in agriculture value chain, with men more engaged in production and physically intensive tasks, while women are more engaged in distributional tasks.

**Table 2:** Gender share of work activities.

Activity	Total	Male		Female	
		Freq	%	Freq	%
Farmer (own or family farm)	8548	5220	61.1	3328	38.9
Agricultural laborer	196	150	76.5	46	23.5
Retail trade (street vendor)	1221	313	25.6	908	74.4
Wholesale and shop-keepers (warehouse)	931	318	34.2	613	65.8
Others	4433	2493	56.2	1940	43.8

Table 3 shows how these activities are distributed across the regions. Farming is almost evenly distributed in the south, with slightly higher participation from women (53.1%) compared to men (46.9%). However, in the North, farming is predominantly by men (68.6%), with women having the lower share (31.4%). In the same vein, agricultural labor is slightly dominated by women in the South but is highly dominated by men in the North. However, in trade-related roles, women are more concentrated in both retailing (81.5% and 68.9%) and wholesaling (75.6% and 55.7%) in both regions, although more pronounced in the South. The “Others” category includes all non–agricultural and non–agricultural-trade occupations reported in the survey. These include paid employment (civil service, teaching, security services), skilled and semi-skilled trades (tailoring, welding, carpentry), transport work, construction labor, domestic services, and self-employment outside food markets.

**Table 3:** Gender share of work activities by region.

Activity	South				North			
	Male		Female		Male		Female	
	Freq	%	Freq	%	Freq	%	Freq	%
Farmer (own or family farm)	1397	46.9	1581	53.1	3823	68.6	1747	31.4
Agricultural laborer	25	48.1	27	51.9	125	86.8	19	13.2
Retail trade (street vendor)	98	18.5	432	81.5	215	31.1	476	68.9
Wholesale and shop-keepers (warehouse)	116	24.4	359	75.6	202	44.3	254	55.7
Others	1072	59.9	719	40.1	1546	44.0	1970	56.0

Table 4 shows the extent of women’s engagement in each activity of the value chain. The table shows that overall, women are most engaged in farming with 58.7%, although the extent varies across the sub-regions, with highest involvement in the North Central (64.6%), moderate in the Core South—which comprises the South-South and South-East geo-political zones, (59.9%), and least in the Core North-made up of the North-West and North-East zones, (36.3%) and the South West (21.2%) respectively. Very few persons, especially women, engage in agricultural labor in all sub-regions as the shares are not greater than 1%. (Table 2 shows farm-labor as 1.3% of reported activities, i.e., 196/[8548+196+1221+931+4433]). Women in Nigeria’s South-West zone are more engaged in trade-related activities in the value chain, as 19.9% women are seen in retailing and 21.4% in wholesaling. The Core North and Core South also see significant share of women in trade with 14.3% in retailing and 7.4% in wholesale in the Core North, while 12% and 8.4% of women are seen in Core South.

**Table 4:** Women’s activity engagement by sub-regions.

Activity	Core North		Core South		North Central		South West		Overall	
	Freq	%	Freq	%	Freq	%	Freq	%	Freq	%
Farmer (own or family farm)	842	36.3	1423	59.9	905	64.6	158	21.2	3328	48.7
Agricultural laborer	5	0.2	24	1.0	14	1.0	3	0.4	46	0.7
Retail trade (street vendor)	331	14.3	284	12.0	145	10.4	148	19.9	908	13.3
Wholesale and shop-keepers (warehouse)	172	7.4	200	8.4	82	5.9	159	21.4	613	9.0
Others	967	41.7	443	18.7	254	18.1	276	37.1	1940	28.4
Total	2317	100.0	2374	100.0	1401	100.0	744	100.0	6835	100.0

### Regression analysis

We used regression models to estimate gender differences in participation. To document differences in consumption, as an indicator or welfare, we used household-level consumption expenditure. (Individual-level consumption data is not available). The logit regression estimates in our analysis were reported as odds ratios to simplify the interpretation of the results.

The logit regression from Table 5 provides evidence on the likelihood of participation in activities. The result shows that women are 49% less likely to participate in farming compared to men, on average (p < .05). However, women are 35% (p < .01) and 189% (p < .05) more likely than men to participate in wholesale and retail activities respectively. This is consistent with the descriptive statistics in the Descriptive Statistics Section, which show men over-represented in farming, relative to their share of the population. Individuals in rural areas are 164% more likely to participate in farming (p < .05), 69% less likely to participate in wholesale (p < .05) and 58% less likely to participate in retail (p < .05). This reflects the structural divide within agricultural production, which is largely in rural, and trade in urban Nigeria. The interaction term shows that rural women are 60% (p < .05) more inclined to diversify into trading activities, pointing to trade as a key livelihood strategy for women in rural settings. For the age variable in Table 5, relative to individuals under 35 years, those aged 35–50 are 33% less likely to participate in wholesaling (p < 0.05) and 29% less likely to participate in retailing (p < 0.05), while showing no statistically significant difference for farming. Individuals aged 51–65 are 45% less likely to participate in wholesaling (p < 0.01), with no significant differences in farming or retailing. In contrast, individuals aged 65 and above are 65% more likely to participate in farming (p < 0.01), while their participation in wholesaling and retailing does not differ significantly from the under-35 group.

**Table 5:** Logit regression of the likelihood of engaging in various activities.

	*Dependent variable: Activity*		
	Farming	Wholesaling	Retailing
	(1)	(2)	(3)
**GENDER**			
Female	–0.68^***^	0.30^*^	1.06^***^
	(0.10)	(0.17)	(0.16)
**SECTOR**			
Rural	0.97^***^	–1.17^***^	–0.87^***^
	(0.07)	(0.13)	(0.13)
**Age group**			
35–50	–0.06	–0.40^**^	–0.34^*^
	(0.08)	(0.17)	(0.17)
51–65	0.12	–0.60^***^	–0.10
	(0.09)	(0.20)	(0.18)
Above 65	0.50^***^	–0.12	–0.32
	(0.13)	(0.25)	(0.28)
**Ever attended school**			
No	0.32^***^	–0.48^***^	0.07
	(0.05)	(0.12)	(0.09)
**Marital status**			
Married	–0.30^***^	0.33^**^	0.22^*^
	(0.08)	(0.13)	(0.11)
Never married	0.19^*^	–0.30^*^	–0.19
	(0.10)	(0.18)	(0.15)
**Own land**			
Yes	3.35^***^	–0.89^***^	–0.76^***^
	(0.18)	(0.11)	(0.10)
**Zone**			
North East	–0.56^***^	–0.43^***^	0.37^***^
	(0.06)	(0.16)	(0.12)
North West	–1.32^***^	1.01^***^	0.79^***^
	(0.06)	(0.12)	(0.11)
South East	–0.23^***^	–0.06	0.49^***^
	(0.07)	(0.15)	(0.12)
South South	–0.50^***^	0.49^***^	0.07
	(0.07)	(0.14)	(0.13)
South West	–0.84^***^	0.50^***^	0.08
	(0.09)	(0.14)	(0.13)
**Own house**			
Yes	0.05	0.18^*^	–0.18^**^
	(0.05)	(0.09)	(0.08)
**Interactions**			
Female × Rural	0.06	0.47^***^	0.16
	(0.10)	(0.16)	(0.15)
Female × 35–50	0.06	0.48^**^	0.48^***^
	(0.09)	(0.19)	(0.18)
Female × 51–65	–0.02	0.85^***^	0.13
	(0.12)	(0.23)	(0.21)
Female × Above 65	0.05	0.13	0.29
	(0.18)	(0.33)	(0.33)
Constant	–2.92^***^	–1.92^***^	–2.10^***^
	(0.22)	(0.24)	(0.22)
Observations	13,104	13,104	13,104
Log Likelihood	–7,623.40	–2,765.77	–3,365.14

*Note:* ^*^p < 0.1; ^**^p < 0.05; ^***^p < 0.01.

Education also appear to play a role in determining entry into the value chain. Individuals with no education are 38% (p < .05) more likely to go into farming, but are also 38% (p < .05) less likely to trade. Education can thus be seen as providing the required human capital for going into trade-related activities, relative to farming, while increasing the odds of entering other professions relative to retail or wholesale trade. Marriage and home ownership, particularly for women, appears to provide additional household resources and networks that can be leveraged to establish small-scale trading activities. This is evident from our result, as married individuals are 26% (p < .05) less likely to go into farming, but 39% (p < .05) more likely to go into trade (wholesale). As expected, people who own land are 335% (28.6 times odds) more likely to farm (p < .05), and about 80% less (0.41 times odds) of trading.

Regional differences reveal the heterogeneity of gender participation in Nigeria’s sub-regions. In the North East (43% (p < .05)) and the North West (73% (p < .05)), individuals are less likely to farm but more likely to engage in retailing compared to the north central. By contrast, in southern regions, women are more heavily engaged in both farming and trading. This means that gendered divisions of labor are not uniform, but are mediated by geography, culture, and local economic structures.

In Table 6, we disaggregate the logit regression by region (North vs. South) which is helpful to show the regional contrast of the variables. From the table, we show that the odds of women participating in farming is lower in the North (47%) (p < .05) as well as the South (45%) (p < .05), while the odds of participating in trade (wholesale and retail) is higher for women, compared to men. This means that women, irrespective of the sub-national region, are less likely to be involved in agricultural production (farming) compared to men, while they are more likely, net of region, to engage in agricultural trade (wholesale and retail), although with higher odds in the south (3.3 times more likely than men to wholesale, and 5 times more likely than men to retail). Both effects are statistically significant (p < .05). Similarly, in both regions, rural dwellers are more likely to farm, with rural dwellers in the north 3.6 times (p < .05) more likely than urban dwellers, and rural dwellers 1.63 times (p < .05) more likely than urban dwellers in the south. Although rural dwellers in both regions are less likely to get involved in trades, people who dwell in rural areas of northern Nigeria are much lesser likely to participate in wholesale (84% p < .05), and retail (67% p < .05). This means rural residence status boosts farming odds dramatically (3.6x in the North; 1.6x in the South), but sharply reduces the odds of participating in wholesale and retail activities.

**Table 6:** Logit regression of the likelihood of engaging in various activities by region.

	*Dependent variable: Activity*						
	North			South		
	Farming	Wholesaling	Retailing	Farming	Wholesaling	Retailing
	(1)	(2)	(3)	(4)	(5)	(6)
**GENDER**						
Female	–0.63^***^	0.01	0.93^***^	–0.60^***^	1.21^***^	1.61^***^
	(0.14)	(0.17)	(0.17)	(0.11)	(0.17)	(0.18)
**SECTOR**						
Rural	1.28^***^	–1.82^***^	–1.10^***^	0.49^***^	–0.51^**^	–0.47^**^
	(0.09)	(0.16)	(0.17)	(0.10)	(0.21)	(0.22)
**Age group**						
35–50	0.14^*^	–0.46^***^	–0.17	–0.22^**^	0.37^**^	0.32^**^
	(0.08)	(0.14)	(0.12)	(0.10)	(0.16)	(0.15)
51–65	0.28^***^	–0.42^**^	–0.26^*^	–0.08	0.35^*^	0.35^**^
	(0.09)	(0.17)	(0.15)	(0.11)	(0.19)	(0.18)
Above 65	0.44^***^	–0.22	–0.43	0.54^***^	0.28	0.19
	(0.14)	(0.26)	(0.27)	(0.15)	(0.26)	(0.24)
**Ever attended school**						
No	0.59^***^	–0.59^***^	–0.10	0.19^*^	–0.70^***^	0.20
	(0.06)	(0.13)	(0.10)	(0.11)	(0.24)	(0.17)
**Marital status**						
Married	–0.39^***^	0.02	0.20	–0.13	0.41^**^	0.30^**^
	(0.12)	(0.21)	(0.18)	(0.10)	(0.16)	(0.14)
Never married	0.35^**^	–0.99^***^	–0.32	0.15	0.13	0.00
	(0.14)	(0.26)	(0.22)	(0.14)	(0.24)	(0.22)
**Own land**						
Yes	3.23^***^	–0.48^***^	–0.73^***^	3.32^***^	–1.01^***^	–0.68^***^
	(0.31)	(0.18)	(0.16)	(0.23)	(0.13)	(0.13)
**Own house**						
Yes	–0.11	0.41^***^	–0.10	0.17^**^	–0.02	–0.17
	(0.08)	(0.16)	(0.13)	(0.07)	(0.12)	(0.11)
**Interactions**						
Female × Rural	–0.22	0.89^***^	0.39^*^	0.77^***^	–0.05	–0.11
	(0.15)	(0.21)	(0.20)	(0.13)	(0.24)	(0.24)
Constant	–3.66^***^	–1.14^***^	–1.44^***^	–3.40^***^	–2.55^***^	–2.80^***^
	(0.35)	(0.32)	(0.28)	(0.27)	(0.27)	(0.26)
Observations	7,908	7,908	7,908	5,196	5,196	5,196
Log Likelihood	–4,757.39	–1,487.90	–1,914.98	–3,037.44	–1,333.16	–1,474.15

*Note:* ^*^p < 0.1; ^**^p < 0.05; ^***^p < 0.01.

From the age variables, an increase in the age of individuals in the north is associated with an increased odds of participating in farming (32% for 51-65, and 55% for above 65). However, in the south, old age increases farming (75% for “above 65”) and also increases the odds of participating in wholesale and retail. In the north, individuals between 35 and 50 years of age are 37% less likely than individuals below 35 years of age to engage in wholesale, however, are 45% more likely in the south.

Education shapes where people enter in value chain in both regions. Although having no education in the north and south channel individuals into farming, the effect is more pronounced in the north (80% less odds) than in the south (21% less odds). In both regions, education opens the access to participate in trades, the effect is slightly higher in the south. Individuals who are married are 32% (p < .05) less likely to participate in farming in the north, but 51% (p < .05) and 31% (p < .05) more likely to participate in wholesaling and retailing, respectively, compared to other marital statuses. Land ownership on the other hand is the single strongest predictor of farming participation in both North and South, with odds ratios above 25 (p < .05). This shows that farming is overwhelmingly tied to asset ownership. Conversely, land ownership significantly reduces the odds of participation in wholesale and retail trade, suggesting that non-landholders diversify into trade as an alternative. Interaction effects confirm that being female increases the likelihood of engaging in trade when combined with rural residence. In both regions, rural women are significantly more likely than rural men to engage in retailing and wholesaling. This means that trade serves as a key livelihood pathway for women in rural areas.

The highest average expenditures recorded among households engaged solely in wholesaling (25,647.59) and those solely in retailing (21,017.73), while farmers only households have the lowest spending (11,926.80). Mixed-activity households, such as those combining farming, wholesaling, and retailing (21,482.93) or farmers and wholesalers (18,939.23), show relatively high expenditures, indicating that diversification into trade raises household spending capacity.

The OLS estimates from Table 7 indicate substantial differences in household spending across activity groups. The baseline category captures households engaged in other activities outside of the three in focus. Households that rely only on farming spend significantly less, with consumption expenditures lower by about 7,210 units–the equivalent of about 6 per week or about 40% of the average household spending, compared to other groups (p < .05). Similarly, households combining farming and retailing also spend significantly less (–4,060) (p < .05), implying that farming households, whether specialized or diversified into retail, face constraints that reflect in their spending. In contrast, households engaged solely in wholesaling spend significantly more (+6,511) (p < .05), showing the higher returns associated with wholesale trade. Retailers alone and other mixed groups (farmers with wholesalers, wholesalers with retailers, or those engaged in all three activities) do not show significant differences under OLS, suggesting that without addressing endogeneity, the effect of diversification is less clear.

**Table 7:** OLS and IV regressions: total household spending vs. activities.

	*Dependent variable: Total spending*			
	OLS	Instrumental variable		
	Overall (1)	Overall (2)	North (3)	South (4)
Farmers only	–7,209.82^***^	–6,836.79^***^	–9,707.81^***^	–4,991.33^**^
	(865.04)	(1,780.28)	(2,887.16)	(2,346.01)
Retailers only	1,881.10	11,325.99^***^	16,237.47^***^	9,422.53^**^
	(1,320.47)	(3,262.84)	(5,768.79)	(3,977.97)
Wholesalers only	6,510.97^***^	11,896.96^***^	21,539.39^***^	8,190.71^*^
	(1,332.63)	(3,437.86)	(6,119.97)	(4,223.99)
Farmers and retailers	–4,059.57^***^	–9,499.13^***^	–7,617.07^**^	–13,986.00^***^
	(1,139.09)	(2,450.55)	(3,851.87)	(3,333.78)
Farmers and wholesalers	–197.39	5,724.65^*^	15,026.99^***^	–603.17
	(1,299.14)	(2,966.47)	(4,782.47)	(3,888.43)
Wholesalers and retailers	–166.96	13,701.66^*^	4,589.03	3,293.10
	(2,824.43)	(7,175.45)	(9,430.33)	(14,812.13)
Farmers, wholesalers, and retailers	2,346.31	1,305.67	–26,655.65^***^	61,449.89^***^
	(2,422.92)	(6,599.71)	(8,780.59)	(14,491.80)
Constant	19,136.63^***^	17,999.09^***^	19,838.44^***^	17,469.68^***^
	(776.75)	(1,637.60)	(2,697.86)	(2,096.28)
Observations	4,305	4,305	2,249	2,056
R^2^	0.05	0.01	–0.06	–0.06
Adjusted R^2^	0.05	0.011	–0.06	–0.06

*Note:* ^*^p < 0.1; ^**^p < 0.05; ^***^p < 0.01.

The IV estimates (from columns 2 to 4) provide a clearer picture. Farming-only households still spend significantly less (–6,837) (p < .05), and the negative effect is even larger when farming is combined with retailing (–9,499) (p < .05). By contrast, households engaged solely in retailing (+11,326) (p < .05) or wholesaling (+11,897) (p < .05) see large, positive, and significant effects on consumption spending, reinforcing that trade-based households have stronger purchasing power.

Disaggregating by region reveals important heterogeneity. In the North (column 3), farming households exhibit sharp spending disadvantages: farmers only (–9,708) (p < .05) and farmers combined with retail (–7,617) (p < .05) both spend significantly less. By contrast, retailers only (+16,237) (p < .05), wholesalers only (+21,539) (p < .05), and farmers with wholesalers (+15,027) (p < .05) show strong spending advantages, suggesting trade is far more rewarding in the north. The South displays a slightly different pattern: farmers also spend less when combined with retail (–13,986) (p < .05), but both retailers only (+9,423) and wholesalers only (+8,191) maintain significantly higher spending, indicating that trade is universally advantageous. What stands out is the large positive effect for households combining all three activities (+61,450) (p < .05), which is unique to the South, suggesting that diversified participation across farm, wholesale, and retail activities is especially rewarding in this region.

To assess the strength and validity of the instruments, we used a series of diagnostic tests for the IV models. The Supplementary Information Section [Supplementary-material pone.0332100.s001] includes a table that shows the details of the post-estimation tests. The tests consistently provide estimates that reject a hypothesis of weak instruments. The Wu-Hausman endogeneity test that we used, also implied that an IV estimator should be preferred for estimation.

## Discussion

Regional differences in gender participation emerge from constraints that shape what men and women do in farm economies. Consistent with prior studies that highlight women’s limited access to land and farm inputs [2,3,20], we find that women are significantly less likely than men to farm. It is reasonable to expect that limited rights to land, lower access to farm inputs, and unequal control over agricultural assets reduce women’s ability to engage in farming [2,20,25,26]. So we explain the results of our analysis showing male-female gaps as feasible, given these barriers, when combined with the demands of unpaid domestic work [27]. Retail and small-scale wholesale trade appear less constrained, as financial entry barriers are lower, time requirements are more flexible, and returns are less tied to land ownership [28]. In our setting, this helps explain why married women and rural women are more likely to appear in trading roles than in farming: trade allows more time-flexibility, especially where markets are nearby and demand for small-scale retail is strong [21,28,29].

Our results show that participating in trade—related activities, especially retailing, wholesaling (especially in the south), are associated with higher household consumption spending. This suggests that women’s participation in distribution and marketing is important to household welfare, in agreement with related papers that link non-farm income to better household outcomes [30]. In our case, retail and wholesale trade appear to offer a parallel welfare-enhancing channel. Age patterns in the results also complement interesting regional differences, with one commonality, a return to farming for those aged 65 and above. Older adults may be expected to turn to work that relies more on the social networks needed to hire farm labor and own landed property. On the other hand, it is useful to consider that other studies showing less financial inclusion in the northern regions of Nigeria, may help to explain why as respondents get older, they are more likely to undertake trade–which requires financial capital, in the southern parts of the country, and less likely to do so in the northern parts (Table 6). This topic itself should be the subject of another paper, given how we are limited in keeping the scope of this paper focused.

The regional differences in our results reflect how social and institutional conditions, which act more like unwritten policies, shape women’s access to work. In northern Nigeria, cultural expectations about women’s movement and public roles limit the types of activities open to women. Women’s ability to farm at scale or participate in large markets where bargaining and long travel are required can be affected by the aforementioned constraints. The pattern could help to explain why women in northern Nigeria appear mostly in small, local retail rather than in farming or wholesaling. The situation differs in southern Nigeria, where mobility rules are less strict and market systems are more developed. Our estimates of household consumption spending are consistent with the other results: in the North, trade offers one of the few ways women can access higher-income activities, while in the South, combining farming and trade produces the largest gains.

The interaction terms in Tables 5 and 6 add depth to our explanations by showing rural-urban differences in female participation, with more of the higher participation of rural women in farming, coming from southern Nigeria. The regional and rural-urban differences are likely due to social norms on mobility and land access. The options faced by women are more sharply limited outside urban centers. Where markets are close and travel demands are lower, women participate more in small-scale trade. In settings with tighter restrictions, these opportunities narrow.

## Conclusion

We show that gender and regional conditions strongly influence how households participate in agricultural value chains. We use data from Africa’s most populous nation, and show that these choices matter for household welfare–given how consumption spending differs by the main activities households undertake. We find that men continue to dominate farming, while women play a much larger role in retail and wholesale trade. However, these patterns differ across regions. In northern Nigeria, women are more concentrated in small-scale retail, likely due to stricter mobility expectations, limited access to large markets, and weaker support structures that reduce women’s ability to engage in farming and higher-value trading activities. In southern Nigeria, social norms are more flexible, markets are better developed, and women’s trading networks are stronger. These conditions allow women to participate more actively in wholesale and retail trade and, in some households, to combine farming with trade. These structural differences explains our welfare results: farming households have the lowest levels of consumption, while trade-based households show clear spending advantages, especially wholesalers. In the South, combining farming with trade brings even larger gains. These results show that gendered economic opportunities are shaped by regional constraints and cannot be addressed by a single national policy.

Our findings suggest some areas for policy action. Targeted programs for women, including programs focused on financial capital for wholesale and retail trade, could ease some of the constraints women face nationwide. Region-specific measures are also important. Subsidized transport, and other innovative approaches that reduce the cost of physical mobility to markets for women traders in the North could help address the barriers that led to our observed gendered differences in levels of participation. Credit lines and shared storage facilities, are other examples of interventions that could enable women in all regions of the country to scale into higher-value wholesaling.

This research study is most relevant to policy in how it shows regional differences in gendered constraints and welfare outcomes. We suggest, based on the differences across Nigeria, that policies tailored to sub-regions and localities are more likely to be effective compared to a uniform national approach. One limitation of this study is that the data prevent us from observing intra-household decision-making processes or specific mechanisms through which women switch from farming to trade. Our IV strategy, though an improvement in identification, cannot capture all the unobserved community-level constraints. Future work should investigate how bargaining within households shapes activity choice as well as test interventions seeking to relax financial capital, mobility and market-access constraints for women to encourage participation in wholesale trade, retail trade, and agriculture.

## Supporting information

S1 Appendix Post-estimation tests. (PDF)
