# Supplementary material for: Gendered activities: Men farm, women trade, or is it less clear-cut?
Source: PLoS One. 2026 Jan 27;21(1):e0332100. doi: 10.1371/journal.pone.0332100 (PMC12844507; doi:10.1371/journal.pone.0332100)
Supplement: S1 Appendix — (PDF) [file pone.0332100.s001.pdf]

Appendix: Post-Estimation Tests

We adopted post-estimation tests to confirm that the instrument used to identify household activity is both valid and sufficient as a consistent measure of the relationship between activity type and household spending.

Table 1. IV Diagnostic Tests for all Models

Model	Test	df1	df2	Statistic	p-value
Overall	Weak instruments (farmers only)	7	4,297	457.49	0.00
Overall	Weak instruments (retailers only)	7	4,297	192.66	0.00
Overall	Weak instruments (wholesalers only)	7	4,297	214.24	0.00
Overall	Weak instruments (farmers and retailers)	7	4,297	168.53	0.00
Overall	Weak instruments (farmers and wholesalers)	7	4,297	146.64	0.00
Overall	Weak instruments (wholesalers and retailers)	7	4,297	131.42	0.00
Overall	Weak instruments (farmers, wholesalers, retailers)	7	4,297	99.74	0.00
Overall	Wu–Hausman	7	4,290	13.44	0.00
Overall	Sargan	0	–	–	–
North	Weak instruments (farmers only)	7	2,241	239.92	0.00
North	Weak instruments (retailers only)	7	2,241	101.55	0.00
North	Weak instruments (wholesalers only)	7	2,241	100.14	0.00
North	Weak instruments (farmers and retailers)	7	2,241	101.91	0.00
North	Weak instruments (farmers and wholesalers)	7	2,241	78.33	0.00
North	Weak instruments (wholesalers and retailers)	7	2,241	89.35	0.00
North	Weak instruments (farmers, wholesalers, retailers)	7	2,241	50.17	0.00
North	Wu–Hausman	7	2,234	13.67	0.00
North	Sargan	0	–	–	–
South	Weak instruments (farmers only)	7	2,048	199.94	0.00
South	Weak instruments (retailers only)	7	2,048	87.57	0.00
South	Weak instruments (wholesalers only)	7	2,048	103.59	0.00
South	Weak instruments (farmers and retailers)	7	2,048	67.91	0.00
South	Weak instruments (farmers and wholesalers)	7	2,048	67.90	0.00
South	Weak instruments (wholesalers and retailers)	7	2,048	32.28	0.00
South	Weak instruments (farmers, wholesalers, retailers)	7	2,048	44.92	0.00
South	Wu–Hausman	7	2,041	8.42	0.00
South	Sargan	0	–	–	–

Across all models, the first-stage F-statistics exceed the conventional threshold of 10. Most specifications show very strong instruments (F-statistics above 100), with the lowest being 44.92 in the southern sample, which still comfortably exceeds the rule-of-thumb cutoff, indicating that the instruments are strongly correlated with the endogenous household activity variable and therefore not weak.

The Wu–Hausman endogeneity test is statistically significant for the full dataset, as well as for the regional subsamples ($p < 0.01$), implying that observed household activity is endogenous and that the IV estimator is therefore preferred. The models are exactly identified, so the Sargan test cannot be computed, which is expected in the absence of over-identifying restrictions.
